# Resource allocation processes at multilateral organizations working in global health

**DOI:** 10.1093/heapol/czx140

**Published:** 2018-02-05

**Authors:** Y-Ling Chi, Jesse B Bump

**Affiliations:** 1 International Decision Support Initiative, Imperial College London, St Marys Hospital, 10th Floor QEQM Wing, South Wharf Road, W2 1NY, London, UK; 2 Department of Global Health and Population, Harvard TH Chan School of Public Health, 677 Huntington Ave, Boston, MA 02115, USA

**Keywords:** Aid, international health policy, resource allocation, global health

## Abstract

International institutions provide well over US$10 billion in development assistance for health (DAH) annually and between 1990 and 2014, DAH disbursements totaled $458 billion but how do they decide who gets what, and for what purpose? In this article, we explore how allocation decisions were made by the nine convening agencies of the Equitable Access Initiative. We provide clear, plain language descriptions of the complete process from resource mobilization to allocation for the nine multilateral agencies with prominent agendas in global health. Then, through a comparative analysis we illuminate the choices and strategies employed in the nine international institutions. We find that resource allocation in all reviewed institutions follow a similar pattern, which we categorized in a framework of five steps: strategy definition, resource mobilization, eligibility of countries, support type and funds allocation. All the reviewed institutions generate resource allocation decisions through well-structured and fairly complex processes. Variations in those processes seem to reflect differences in institutional principles and goals. However, these processes have serious shortcomings. Technical problems include inadequate flexibility to account for or meet country needs. Although aid effectiveness and value for money are commonly referenced, we find that neither performance nor impact is a major criterion for allocating resources. We found very little formal consideration of the incentives generated by allocation choices. Political issues include non-transparent influence on allocation processes by donors and bureaucrats, and the common practice of earmarking funds to bypass the normal allocation process entirely. Ethical deficiencies include low accountability and transparency at international institutions, and limited participation by affected citizens or their representatives. We find that recipient countries have low influence on allocation processes themselves, although within these processes they have some influence in relatively narrow areas.

Key Messages

- Extensive review of resource allocation at multilateral organisations working on global health finds the existence of well-structured and organized systems.
- Core principles of each institution are reflected in their design choices of allocation cycles (e.g. emphasis on fairness, health needs, efficiency).
- Although processes are documented, confidential or non-transparent elements such as qualitative adjustments and earmarking agreements undermine the transparency and legitimacy of allocation decisions and restrict the knowledge and participation of recipient countries and their citizens.

## Introduction

International institutions provide well over US$10 billion in development assistance for health (DAH) annually, and between 1990 and 2014 DAH disbursements totalled $458 billion (IHME 2014)—but how do they decide who gets what, and for what purpose? The importance of these questions is difficult to overstate for the six billion people in Low- and Middle-Income Countries (LMIC) whose health can be directly affected by DAH. Allocation decisions affect all three pillars of public health: the political, the technical, and the ethical (Roberts *et al*., 2003). Politically, allocation processes determine which countries get what assistance, raising distributional issues at every level from the international down to individuals. Technical matters include the choice of objectives, for instance the diseases to be addressed and the strategies that will be employed, the population to be targeted, and the effectiveness of interventions and programs. Ethical considerations start with the fairness of the allocation processes, and continue through the equity of their outcomes and consequences.

The Equitable Access Initiative (EAI) was designed to explore some of these issues—primarily the technical aspects. The nine convening international agencies were particularly concerned with understanding the consequences of using gross national income (GNI) per capita as a primary indicator of need, and interested in exploring alternatives. Although GNI had long been used for that purpose, they noted that rising inequality means that most of the world’s poor now live in middle-income countries. Hence, the EAI was convened to investigate how processes might better reflect disease burdens, national capacity to intervene, government health budgets and other factors (EAI 2015a, b) primarily the technical aspects such as classifying country needs and capacities in health.

Under the aegis of the EAI, academic teams were engaged to develop fresh thinking on classification methods for capturing country needs and capacities in health, the products of which were the basis for papers in this special issue. These colleagues analysed the determinants of health outcomes to explore the value of GNI per capita as a proxy (Sterck *et al.*), modelled government ability to spend on health under various conditions over time (Haakenstad *et al.*) and conducted experiments to discover the values that participants felt should be prioritized in allocation (Grepin *et al*.) and then applied these ideas to develop new country rankings (Ottersen *et al.*).

As an associated activity, we explored how allocation decisions were made by the EAI’s nine convening agencies themselves—Gavi, the Vaccine Alliance (hereafter Gavi), The Global Fund to fight AIDS, Tuberculosis, and Malaria (GFATM), The United Nations Population Fund (UNFPA), The United Nations Children’s Fund (UNICEF), The United Nations Development Programme (UNDP), The World Bank Group’s International Development Agency, The World Health Organization (WHO), UNAIDS and UNITAID. We felt that examining allocation practices at these prominent institutions would be helpful to discussions about the future of allocation and of great interest to recipient countries and their citizens.

To support our larger objective of fostering greater transparency, more informed discussion, and better allocation, we provide clear, plain language descriptions of the complete process from resource mobilization to allocation for each multilateral. Then, through a comparative analysis we illuminate the choices and strategies employed in the nine international institutions.

Our investigation of allocation methods at international agencies connects to several important literatures in global health, including DAH and allocation, aid effectiveness and ethics. Some have analysed where DAH goes. Long time-series data have been used to analyse the patterns of DAH, but the results of those studies are not conclusive. For instance, Piva and Dodd (2009), Hotez *et al.* (2014) and Dieleman *et al.* (2015) find wide variation in funding across regions and disease areas that are not explained by differences in disease burden or income. Estimation problems stemming from model misspecification, unobserved variables and measurement problems have constrained empirical approaches (McGillivray 2003; Hoeffler and Outram 2008). Others have discussed the politics of DAH, particularly the haphazard process in which issues rise and fall on the global health agenda (Shiffman & Smith, 2007; Hafner & Shiffman, 2012; Parkhurst & Vulimiri, 2013; Reich, 1995; Bump, Reich, & Johnson, 2013).

Aid effectiveness has attracted lively commentary, attention sufficient to produce The Paris Declaration on Aid Effectiveness (2005), and a robust literature, if not yet a consensus answer (Bourguignon & Sundberg, 2007; Easterly, 2008; Piva and Dodd 2009; Hristos and Paldam 2009; Clemens *et al.* 2011). Adequate and transparent resource allocation systems are often mentioned as desirable, for instance as Point 17 in the Paris Declaration, but as far as we were able to detect, only Ottersen *et al.* (2014) have directly addressed the question of allocation criteria. Their work details the criteria in allocation formulae in 10 bilateral and 5 multilateral institutions working in global health and discusses their distributional impact. Our aims differ in that we seek to understand the complete decision-making process leading to allocation decisions. Moreover, unlike previous works, we supplemented a review of literature with interviews at each institution.

There is a very large literature on distributive justice and other aspects of ethics applied to global health. Much of this has centred on priority setting, which can be taken loosely as a synonym for allocation in this context. Scholars have emphasized ‘accountability for reasonableness’ in such decisions (Daniels 2000, 2007), the importance of ‘making fair choices’ of what to provide to whom in health (Ottersen and Norheim 2014), and the centrality of ethics in all public health rationing decisions (Roberts and Reich 2002). Our inquiry sheds light on an important aspect of priority setting—how international agencies allocate DAH.

We organize this article as follows. Section 2 The following section presents our methods and data. Section 3 contains a descriptive table of the allocation process in each institution and summarizes our comparative analysis. Section 4 provides discussion and conclusions.

## Methods and data

### Institutions for analysis

Since our project was linked to the EAI, we focussed our analysis on its convening agencies—Gavi, GFATM, UNFPA, UNICEF, UNDP, the World Bank’s IDA, WHO, UNAIDS (meaning the UNAIDS Secretariat) and UNITAID. Each of these institutions has significant objectives in global health and a worldwide mandate, as we summarize in Table 1. At present there are no other large international institutions meeting these two criteria. Regional development banks, bilateral agencies and foundations are also important objects of study, but these were not considered in the EAI and were beyond the scope of this article. Collectively, the EAI convening agencies provided US $11.7 billion in DAH in 2014 (UNITAID 2013; Institute for Health Metrics and Evaluation 2014). In that year, the largest sources were GFATM, WHO and Gavi (respectively US $4.1 billion, US$2.1 billion and US$1.8 billion).

**Table 1:** Institutional objectives

Institution	Objectives
Gavi	Save children’s lives and protecting people’s health by increasing access to immunisation in poor countries
GFATM	Accelerate the end of HIV/AIDS, tuberculosis, and malaria as epidemics
UNAIDS	Formulate a plan of action for all the institutions working on HIV/AIDS to end the epidemic by 2030
UNDP	Help eradicate poverty and reduce inequalities and exclusion
UNFPA	Deliver a world where every pregnancy is wanted, every birth is safe, and every young person’s potential is fulfilled
UNICEF	Promote the rights and wellbeing of every child
UNITAID	Contribute to scale-up access to treatment for HIV/AIDS, malaria, and TB for people in developing countries by leveraging price reductions of quality drugs and diagnosis
WHO	The attainment of the highest possible standard of health by all people.
World Bank	End extreme poverty by decreasing the percentage of people living on less than $1.25 a day to less than 3%; promote shared prosperity by fostering the income growth of the bottom 40% for every country

### Data collection

In March 2015, we conducted a literature review on resource allocation in multilateral organizations using Google Scholar and the keywords ‘resource allocation” combined with either ‘multilateral organisations’ or and institution name, e.g. ‘Gavi.’ To capture grey literature we repeated the search using generic Google and browsing the publication section of each institution’s website. The results included board decisions, budget documents, financial reports, reports on replenishments and other public documents. Documents from 1998 to 2015 were included in this review.

We found that all institutions published some information on allocation, although public disclosure varied substantially over the sample. To complement this literature search, for each institution we conducted semi-structured interviews with at least two senior managers working on resource allocation or related policies or processes (for instance heads of policy, senior programme managers, directors of data and information and the like) between April and November 2015. The interviews lasted ∼1 h, and were conducted in person for the five institutions headquartered in Geneva, and by phone or Skype for the remaining four institutions. For each one, we began by asking for a succinct account of the main resource allocation steps without providing further guidance. After the interviews we compared notes to produce a consensus account. Where there were uncertainties or we found discrepancies with published sources we asked additional questions. We then provided the completed account to each interviewee for verification, making additional adjustments, if needed.

### Framework for analysis

**Figure 1:**
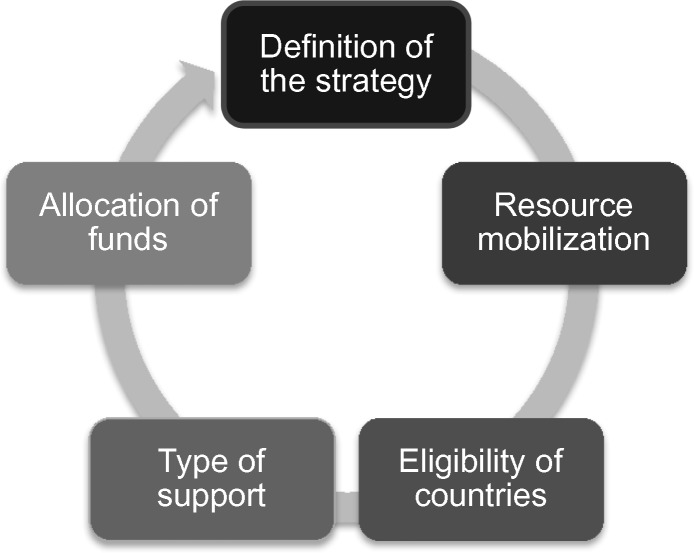
Allocation cycle in multilateral organizations

To structure our analysis we developed a model allocation cycle based on five common themes that we judged to be prominent in our review of documents and in our interviews, presented below as Figure 1. The first of the five steps is the definition of institutional strategy, in which its governing body and/or senior leadership decide organizational goals. The second step is resource mobilization. From there, we identified the sequence of decisions that lead to allocations. Step 3 is an eligibility determination; step 4 (type of support) is the determination of what funds, services, in kind-support or other resources will be made available; step 5 is the allocation of specific resources to specific recipients, such as programmes or countries. Based on our document review and interview transcripts, we categorized the collected information following this framework of analysis. For each step, we documented the institutional mechanism and gathered information on the actor(s) responsible.

We recognize that in practice the five steps do not necessarily occur in order and are not always easily distinguished from one another. For instance, the determination of the strategy is connected very closely to resource mobilization; typically these two things happen in dialogue with one another. Further, some of these steps are negotiated on different cycles, as with replenishment activities or during programmatic strategy reviews. Nonetheless, we believe this model is useful for clarifying the elements of the allocation process and facilitating a comparison across the institutions we study.

### Limitations

Our study relies mainly on documentary evidence as made available by the institutions in our sample and on information collected during our interviews and literature search. As a result, this approach presents several limitations. First of all, it is possible that interviewees overlooked important information, consciously or by mistake; or didn’t consider informal processes that shape allocation decisions. We could not gather information about the politics that could lead to deviations from the formal processes as neither author had intimate access to board discussions or diplomatic channels. In addition, we might not have discovered all aspects of resource allocation in the interview and review process because we did not ask the right questions or find the appropriate documents in our literature search process. We also do not know how much of the process is shaped by the decisions that we attempted to document. It is possible that very large amounts of resources are not accounted for in official documents, and are allocated through undocumented parallel systems.

Finally, our study was conducted mainly in 2015, but allocation processes are dynamic and in some cases were already changing by the time this article was drafted. Where we are aware of such changes we say so in the text, but in none of the cases do we intend to suggest that processes will remain as we found them in 2015.

## Results and discussion

The resource allocation cycle was used to provide a summary of the allocation cycle in each institution (Table 2), as well as to structure the discussion on the trends across our group of institutions (below). For further information about individual institutions, as well as the corresponding references, an extended account is available in Supplementary Annex.

**Table 2:** Summary description of resource allocation cycles

	Strategy	Resource mobilization	Eligibility of countries	Type of support	Allocation of funds
Gavi	Formulated every four years by the Executive Board (includes Gavi’s CEO, 28 members of international organizations, independent experts, vaccine industry)	Replenishment after the strategy is defined and adopted, resources are mobilized during a pledging conference. Between 2000 and 2014: 69% from direct contributions from governments, foundations and private donors. 31% generated through innovative financing mechanisms. Gavi’s funding is mainly received in the form of open contributions (i.e. not earmarked)	Only countries with a GNI per capita below US$1580 + DPT3 immunization rate above 70% of the eligible population. Countries have to apply for support (three windows of application per year), and the independent Review Committee and the Gavi Secretariat decide on the outcome of the application. Countries benefit from support until they are no longer eligible (*e.g.* GNI pc exceeds threshold)	- Funding of 11 vaccine programmes (in current vaccine portfolio) - Technical support for health system strengthening (HSS)	The resources are allocated separately for health systems support and vaccine programmes. For HSS, size of annual birth cohort is multiplied by $5 for countries with a GNI per capita lower than $365, and by $2.5 for countries with a GNI per capita comprised between $365 and $1580. For vaccine programmes, the size of the birth cohort is multiplied by the price of the vaccine minus the level of co-financing. Level of co-financing is based on country’s income (countries with an income below $1045 in 2015 contributed $0.20 per vaccine dose). In addition, for newly approved vaccine programmes, a vaccine introduction grant is available ($0.80 per child in the birth cohort for all vaccines except Human papilloma virus vaccine, for which countries receive $2.40 per girl in the birth cohort)
GFATM	New strategy defined every four years by the Strategic Investment and Impact Committee, led by the Executive Director, strategy leads, and consultants.	Replenishment after the strategy is defined and adopted, resources are mobilized during a pledging conference. 95% of GFATM funding comes from national governments and the European Union. The remaining share of funding comes from private foundations, corporations, and faith based organizations (5%). The GFATM receives very limited earmarked funding.	The GFATM applies income-based and disease-burden based eligibility criteria. High-income countries are not eligible, regardless of disease burden. Depending on income, further eligibility criteria are applied (e.g. focus of application, counterpart financing, G-20 membership). Countries have to apply for funding, with the support of the Secretariat through the Country Coordination Mechanism, and validated by the Technical Review Panel.	The GFATM supports countries in three disease areas (HIV/AIDS, Malaria, and Tuberculosis). Countries can also apply for HSS support.	The bulk of resources is allocated through an allocation formula, except for countries with higher income and lower disease burden. For those countries, allocations are calculated using population size. In addition, funds are set aside for innovative projects. Allocation is calculated every four years following the replenishment. Allocations are calculated using an allocation formula that relies mainly on a country’s disease burden (indicators selected through consultation with partner expert organizations) and country’s ability to pay (which is derived from the GNI per capita). Countries receive a score from the multiplication of these two elements, which corresponds to the country share, relative to the total funding envelope. Allocations are further adjusted using the following indicators: performance, impact, increasing rates of infection, absorptive capacity and other considerations. In addition, minimum and maximum caps are applied
IDA/WB	The strategic development of the institution is discussed on a three year cycle, but a ‘strategy’ is not thoroughly developed. This discussion takes place during a meeting with 52 IDA Deputies and 10 borrowing representatives. IDA Deputies are appointed from the member states of the WB	Every three years, resources are replenished through a large pledging conference. IDA’s resources come from loan repayment, income generated by other parts of the WB Group, and contributions made exclusively by governments. IDA does not receive earmarked contributions	Only works with low-income countries, although some middle-income countries with poor credit ratings exceptionally benefit from support. The eligibility for support is therefore defined by two criteria: income (threshold set at $1205) and lack of access to capital markets. Countries also need to be part of the International Monetary Fund. To access funding, countries need to undergo a systematic assessment and a country partnership framework is defined to decide on the use of funds	Loans to finance development activities, at a zero interest rate and with a grant element depending on country’s risk of debt distress	After each replenishment, a resource envelope is defined for all eligible countries. This resource envelope is allocated based on an allocation formula (Performance Based Allocation) that includes the following indicators: country performance rating, population and GNI per capita. The most important component is the country performance rating, which is itself defined using the Country Policy and Institutional Assessment (CPIA) and the Country Portfolio Performance score, which is calculated using the occurrence of problems in previous IDA loans. Allocations are adjusted as follow: a minimum allocation is set (4 million SDR), as well as a maximum allocation. A grant element is defined based on a country’s risk of debt distress (for countries in high risk, the allocation is provided entirely in the form of a grant). Countries undergo a systematic assessment to determine the constraints and opportunities to growth and poverty alleviation. Based on this assessment, countries formulate an application that details the programmes and interventions to be supported by the loans
UNAIDS	The strategy defines a set of coherent activities and ‘results areas’ for UNAIDS and its co-sponsors organizations. It is approved by UNAIDS’ governing body, the Programme Coordinating Board (PCB), which includes representatives of 22 countries, the 11 cosponsors, and 5 NGOs.	Contributions are raised from governments, Co-sponsors, private partners and foundations through an on-going financing dialogue. National governments contribute 94% of the UNAIDS budget, followed by 3% from Cosponsors, and 3% from other partners (including private sector partners and foundations).	UNAIDS does not allocate funds to countries but to Cosponsor organizations. There are 11 Cosponsors (10 UN organizations and the World Bank).	Supports the work of Cosponsors on HIV/AIDS to ensure that the global response to HIV/AIDS is coordinated.	About one-third of the resources are allocated to cosponsors to strengthen their own programmes and resource mobilization for HIV/AIDS. The remaining two-thirds of resources are used for ‘development activities’ conducted by the Secretariat. Funding allocated to each cosponsor is determined based on “epidemic priorities, performance of the Cosponsors and the funds that each Cosponsor raises” (UNAIDS, 2011)
UNDP	Every four years the Administrator of UNDP and the Executive Board supervise the development of a new strategy. The Executive Board is jointly shared with UNOPS and UNFPA and composed of member countries representatives.	A budget is prepared following each strategy, and resources are mobilized from on-going fund raising. Funding mainly come from governments, other multilateral organizations and private organizations. 74% of contributions are earmarked to specific activities. Only 26% of resources are core resources.	All countries are eligible for support, except those with a GNI per capita of $12 475 or more. Resources are allocated to country offices.	Country Policy Support is available to all countries where need is perceived Service delivery through its offices Research	Core resources (26% of all resources) are divided into three tiers under Targets for Resource Assignment from the Core (TRAC) (UNDP, 2013a): TRAC-1 (60% of regular funds) for programs TRAC-2 (31% of regular funds) is a flexible fund that rewards projects that are well performing, high impact, or innovative TRAC-3 (8% of regular funds) for conflicts or emergencies, such as natural disasters TRAC-1 resources are allocated using GNI per capita and total population. Allocation are raised if a country is categorized as an LDC (UN definition). Allocations are at least $350 000 to $500 000 depending on whether they have a country office. Additionally, UNDP applies the following targets: 85 to 91 per cent of resources should be disbursed to low-income countries, 9 to 15 per cent to middle-income countries and at least 60 per cent to LDCs. Core resource allocations are complemented using non-core resources that are raised specifically to fund country programmes. These resources are raised based on the UNDAF.
UNFPA	Prepared every four years, and approved by the Executive Board (see cell above).	From the strategy, the budget for the institution as a whole is determined. Resources are collected through an on-going fund raising process. Resources mainly come from voluntary contributions from governments, non-governmental organizations, foundations, and private institutions. 53% of collected resources are earmarked to a specific activity or a thematic fund. The non-earmarked funds, 47% of total contributions, are pooled as core resources (often referred to as ‘regular resources’)	UNFPA works with all countries based on an engagement framework that reflects country needs and domestic financing abilities (ranging from policy dialogue and advocacy to service delivery and interventions (in low-income countries with needs judged high or very high). Resources are allocated to country offices.	Policy and advocacy Capacity development Research Norms and standards setting Interventions, and technical support	Regular resources are allocated through three systems: (i) the Resource Allocation System (RAS) (ii) global and regional programme and (iii) institutional budget. The RAS assesses country needs using the following six indicators: (i) skilled birth attendance for the poorest quintile of the population, (ii) proportion of met demand for modern contraception, (iii) adolescent fertility rate, (iv) maternal mortality ratio, (v) Gender Inequality Index, (vi) HIV prevalence among 15–24 year olds. In addition, needs take into account the risk of humanitarian crises and inequality. Countries are grouped in four categories of need. Each category of countries (based on needs) receives a share of the total resources, which is set during the definition of UNFPA’s strategy. For instance, countries with the highest needs and lower income receive 53–63% of the total envelope, whereas countries with the lowest needs and highest income receive between 9–13%. A minimum allocation is also set between $300 000 and $500 000 depending on the income group. UNFPA also receives resources earmarked to one of its thematic fund (*e.g.* maternal health), which are disbursed in a similar fashion. Similarly to UNDP, non-core resources are also raised to fund country programmes as set out in the UNDAF.
UNICEF	New strategy every four years that includes an integrated plan and budget. Strategy is approved by the Executive Board, composed of 36 members elected every three years by the UN Social and Economic Council, and represents all regions.	Resources are raised through on-going financing dialogue based on strategy, budget, and country-specific UNDAF. Governments, non-governmental agencies and foundations contribute to the budget. UNICEF also receives resources raised by national committees (in 36 countries). 26% are core resources; the remainder is earmarked.	Works with all countries where UNDAF assessment indicates need, irrespective of income. Resources are allocated to country offices.	Policy and advocacy Research Norms and standards setting Interventions, and technical support	Core resources are allocated using a formula that includes the following criteria: under five mortality rates, GNI per capita, and the population of children aged 5 or less. For each indicator, countries receive a weight/point ranging from 0 to 1 based on their ranking (*e.g.* 0 attributed to the country with the lowest child mortality, and 1 to the country with the highest). An index is obtained by multiplying all three weights, and is then used to calculate the allocation. The following adjustments to the calculated allocation are made: cap for the difference between two allocation periods (10%), minimum allocation (of $750 000 for all countries but high income), spending target of at least 60% to LDC, and of 50% to Sub-Saharan Africa. Similarly to UNFPA and UNDP, core resources do not represent the majority of resources disbursed. Non-core resources are earmarked resources to specific activities, and they are raised to support specific country programmes (based on the UNDAF).
UNITAID	The strategy is defined every four years by the Executive Board, composed of 12 members, including one member appointed jointly by the five founding governments (Brazil, Chile, France, Norway and the United-Kingdom) and Spain, representatives of the African and Asia region, of civil society, of WHO and of the constituency of foundations.	Raises funding through on-going resource mobilization from national governments and innovative financing. In 2014, 50% of the funding was raised from the solidarity levy on airline tickets, and the remaining contributions were received by national governments and two foundations.	The institution does not work directly with countries, but with implementing partners (*e.g.* UNICEF, GFATM, the Clinton Health Access Initiative). Implementing partners are then in charge of distributing supplies to countries, which are selected based on a dialogue with the Secretariat. In general, at least 85% of funded supplies must be distributed to low-income countries. No more than 10% in lower middle-income countries or 5% to upper middle-income countries.	Provides large scale multi-country grants to improve and accelerate the access of drugs and supplies.	Funding to projects is allocated on a rounds-based system, which starts with a call for proposal. Organizations submit a letter of intent and if approved, a full proposal that includes the description of the equipment, drug or supply, timeline, budget, organisational details, policies on ethics, anti-discrimination and the environment. A proposal review committee and the Secretariat review the proposal, and submit recommendations to the Executive Board.
WHO	The World Health Assembly (composed of 194 member state representatives, usually ministers) meets annually to define and approve the program of work, set major policy directions and approve the budget.	The WHO does not raise funds through on-going resource mobilization efforts. 25% of total resources come from ‘assessed contributions’ paid by member states based on income. Additional funding is raised through a financing dialogue with governments, other UN organisations, other intergovernmental organizations, foundations, NGOS and the private sector. The vast majority of these contributions (93%) are earmarked to specific activities (only 7% are core funding)	All member countries (without regard to income) and work with partners (NGOs, civil society organizations). Resources are allocated to country offices.	Research Norm and standard setting Policy dialogue Programme support Capacity building and technical support Emergency interventions	Resources are allocated to major offices (regional and headquarter), which then allocate to countries every two years, based on life expectancy and GDP per capita. Using these two indicators (which are scaled and multiplied to obtain a country score), countries are ranked into deciles. Country shares are calculated by multiplying the needs index (assigned to each decile) with the log of population squared. Regional allocations are made by aggregating country weightings in a given region. It is worth noting that this resource allocation methodology only applies for core resources. A working group was set up in 2014 to revisit this methodology, and a decision in April 2016 was made to add more indicators and not rank countries into deciles (using the country score instead). In addition to the existing indicators, under-5 mortality and non-communicable disease prevalence, poverty headcount, and indicators of access (health workforce density, political instability, and DTP3 coverage) will be included to calculate allocations for each country

A Supplementary Annex is published with this paper and contains a detailed summary of the process for each institution, as well as all bibliographical information for the information presented in this table.

### Strategy

All of the reviewed institutions had mechanisms for setting organizational strategies, which typically include various goals and specify corresponding activities. In our sample, the strategies were updated on a cycle that ranged from 4 to 7 years. In each case, the highest governing body of the institution held final authority for approving the strategy.

We found no evidence to suggest that the frequency of strategy setting was a significant variable in explaining allocation decisions.

### Resource mobilization

We found that resources are mobilized either through periodic replenishment, as at GFATM, Gavi, and IDA, or via continuous fundraising, as by UNITAID and UN system institutions. For periodic replenishment, donors make multiyear commitments that are pooled, and collectively comprise the budget. As practiced by UNITAID and UN institutions, continuous fundraising engages many sources, including membership fees, voluntary contributions and other activities such as selling products.

We find that these two resource mobilization approaches generate substantially different outcomes in our sample. Institutions employing periodic replenishment exercised complete, or nearly complete autonomy over their own budgets because contributed funds were pooled and not earmarked, except in rare circumstances. But institutions reliant on continuous fundraising received relatively few untied contributions. UNDP, UNICEF and WHO receive around 75% of total resources as earmarked contributions, whose use is negotiated on an ad hoc basis with each donor and subject to whatever restrictions are agreed. These earmarked funds are allocated as negotiated, and are not subject to the normal institutional allocation process.

### Eligibility

The most common eligibility metric was GNI per capita. Five of the seven institutions used a threshold based on GNI per capita, although these were set at different levels to align with different institutional principles. For instance, only countries classified as low-income by the World Bank are eligible for support from Gavi and IDA, which reflects a prioritization of the poorest countries. Gavi then emphasizes absorptive capacity by including vaccine distribution performance requirement as measure of health system capacity. IDA includes measures of access to capital because it wants to channel resources to countries that have the fewest alternatives. GFATM emphasizes health needs and is willing to work with all countries except those classified as high-income. At UNFPA, UNDP and UNICEF, eligibility is also determined through the UNDAF, regardless of income level. Similarly, WHO also works with all countries where need is identified from the Country Cooperation Strategy.

Although ‘eligibility’ implies a binary decision, we find substantial nuance in two dimensions—where engagement is sought, and then what type of support is provided. Among the seven institutions that provide support directly to countries we find that variation in eligibility determinations largely follows different conceptualizations of need (UNAIDS and UNITAID are not covered here). The rules that define eligibility are nevertheless very influential because they do circumscribe subsequent allocation decisions.

### Allocation

Resources are allocated to specific programmatic and country activities within the bounds determined by the eligibility decision. Here, we review how these allocation decisions are made by describing the processes that are used and the factors that are considered. We structure our review by first discussing allocation systems at each institution, and in the six cases where there is more than one, how funds are divided between them. Second, we examine the primary variables used in the main allocation system. Third, we discuss adjustments, which may reflect qualitative factors or targets. The fourth step is country engagement, when the results of the allocation process are negotiated to arrive at a final figure.

UNITAID and UNAIDS do not make allocations to countries and follow different processes. UNITAID makes allocations to implementing partners based on submitted proposals, and UNAIDS allocates resources to Co-sponsors via a consultative process that reflects each institution’s mandate, performance and capacity to mobilize resources. We do not discuss these two further (see Supplementary Annex for more details), concentrating instead on the seven institutions that allocate to countries and programmes.

#### Resource allocation systems

Multiple resource allocation systems are used by each institution, with the exception of IDA. GFATM uses a formula and subsequent adjustments to allocate the bulk of its resources. In parallel, ‘innovative and impactful’ projects are funded from the Incentive Quality Fund. Gavi provides support for vaccine programmes through a rounds-based system, and separately, allocates funding for health systems strengthening interventions using a resource allocation formula. In these two cases the use of two allocation systems corresponds to two separate activities. At UNDP, UNFPA, UNICEF and WHO, different systems are used to disburse different types of resources. These institutions use a formula to allocate core resources. Non-core resources—the majority of total resources—are allocated via fragmented and poorly documented processes. This represents a significant limitation for our analysis of allocation systems because the processes we describe are used for only 25–50% of resources for those institutions.

We do not find that the number of systems corresponds to differences in allocation. Similarly, we find no systematic difference between the results of processes that use an allocation formula vs those that use application rounds.

#### Indicators used in the primary resource allocation system

Table 3 provides an overview of indicators used to drive decisions on the largest share of resources for Gavi, GFATM and IDA, and allocation of core resources for institutions of the UN system. In UN system institutions, the primary allocation system is not used for the majority of resources because those are handled according to ad hoc agreements that we were unable to discover.

**Table 3:** Summary of indicators used in allocation formulae

Institution	Types of indicators
Gavi	Size of the birth cohort, price of vaccine, GNI per capita (to calculate co-financing element)
GFATM	Disease burden (calculated separately for each disease), GNI per capita
UNDP	GNI per capita, population size
UNFPA	Skilled birth attendance for the poorest population quintile, proportion of demand for modern contraception satisfied, adolescent fertility rate, maternal mortality ratio, Gender Inequality Index, HIV prevalence in 15–24 year olds, GNI per capita
UNICEF	Under five mortality rate, GNI per capita, and child population
WHO^a^	Life expectancy and GDP per capita
World Bank	CPIA, Country Portfolio Performance, Population size, GNI per capita

a Applicable for the 2016–2017 budget period, but WHO is undergoing a reform of its resource allocation formula for core resources

Our comparison of indicators used for allocation finds that GNI per capita is used by all institutions, although not in the same way or with the same weight (and WHO uses GPD rather than GNI). At GFATM and UNICEF, GNI per capita is considered using a sliding scale that gives more weight to poorer countries and smooths thresholds at higher levels. In contrast, in Gavi, GNI per capita is used to define the co-payment on the vaccine drug ($0.20 per vaccine dose for countries with a GNI per capita below $1045 in 2015). At IDA, GNI per capita is used directly in the allocation formula, although more emphasis is given to Country Performance Ratings (mainly the Country Policy and Institutional Assessment).

Overall, we found that most institutions adjust allocation with some health indicator. This is simplest at WHO, which uses life expectancy, and most complex at UNFPA which uses five health statistics and a gender inequality index in addition to GNI. Only IDA and UNDP do not include a health indicator, although both use population size, and in the case of IDA, indicators of country performance. Surprisingly, we find that effectiveness or results are not included in the primary resource allocation model; despite the widespread concern in those institutions on results or issues such as ‘value for money’.

This step seems to account for a great proportion of the observed differences in resource allocation. We were not able to collect information on how institutions chose those indicators. From this result, we postulate that, to some extent, those indicators are in line with institutional mandate.

#### Types of adjustments and spending targets

Qualitative adjustments are subsequently applied to determine the final allocation figures. Some adjustments are clear, the most common of which were minimum and maximum allocation limits, and caps on variance between successive allocations. In addition, some institutions define spending targets to ensure that allocation decisions are aligned with institutional principles. For instance, UNICEF has a target for programme allocations of 50% to Sub-Saharan Africa and 60% to countries classified as LDCs (UNICEF, 2012b). However, as a few informants explained to us, other adjustments are used to account for important factors that resist quantification, for instance, absorptive capacity, the likelihood of corruption, past performance or current political issues. Those adjustments rely on internal data that are not made public. At UNDP, qualitative adjustments are defined internally and approved by the Administrator. At the GFATM, qualitative adjustments are made to take into account a wide range of considerations including past program performance, risk and absorptive capacity.

In most cases, such qualitative adjustments are not easily characterized, although it is clear from our review that they can be important, as at UNDP where 30% of core resources are allocated this way. We could not document how these adjustments are made or understand in detail what they reflect.

## Conclusion

DAH has a critical impact on health services and health outcomes for many of the world’s people, including the poorest and most vulnerable. Yet how exactly these resources are allocated has escaped too many scholars and citizens. This article was motivated by the conviction that careful and transparent discussion of allocation processes promotes equity and leads to better outcomes. Documenting allocation processes proved difficult and it is possible or even probable that some details we report will require revision. This hazard is created by the absence of transparency on allocation processes and speaks to the value of fostering transparency and public discussion, both of which promote fairness and accountability.

Considering technical factors, we reached mixed conclusions. At all institutions we found systematic allocation processes incorporating many factors; none relied solely on GNI per capita. We also found that allocation formulae represent only a relatively narrow part of the allocation process, which is constrained far more by choices relating to strategy, type of support, eligibility, and qualitative adjustments. Country participation in these aspects is very limited. Allocation decisions cannot be understood by focusing on individual components, such as an allocation formula or the indicators it considers. Even taken as a whole, the formal allocation cycle does not explain all allocation—it is bypassed where earmarked resources are concerned, and subject to post-hoc adjustments, as well. Moreover, despite the existence of many monitoring and evaluation systems, country performance seems to be considered only qualitatively in determining allocations (except at the GFATM and IDA, and at the former the main performance measure is whether previous funds were spent). In a field preoccupied with measurement and effectiveness, this struck us as odd.

We were puzzled also by the lack of attention given to the incentives generated by resource allocation processes. Most institutions emphasize absolute need, meaning that the relationship between need and allocation is positive. At face value, this means that countries are given fewer resources when they perform well, and, in expectation, it would drive resources to ineffective actors and programs—the ones with the greatest need and lowest performance. We find this problematic, especially in countries where DAH accounts for a large share of health resources. Further research should analyse the incentives created by allocation systems and how they might impact country performance in health.

Politically, we find that resource allocation activities are sensitive and have been hidden from view as a result. Not all aspects of the process were made public, and many available descriptions were difficult to decipher. Qualitative adjustments to the results of formulae were commonly mentioned, but very challenging to document. Negotiations around earmarking were not disclosed, either, and such funds are not subject to the normal allocation process at all. At UNICEF, UNDP and WHO, >60% of total funds earmarked. We recognize that confidentiality may be appropriate in stages, but we argue that there should be clarity about all parts of the process, even if the operational details of some steps are kept confidential. We conclude that further efforts are needed to improve the accountability and the transparency of the decisions that shape resource allocation.

From an ethical perspective, this study raises concerns about the representation of countries in resource allocation, which relied almost exclusively on internal institutional processes. In some organizations, countries are consulted individually about different parts of the process, such as potential funding priorities, or implementation details. However, country participation is limited during the wider decision making process. At the GFATM, countries are engaged at the end of the process to negotiate their own allocation, although only at the margin and in exceptional cases. As a result, countries’ expressions of need and inputs seem to have only a limited effect on how overall funding envelopes are split between activities or countries. A resource allocation cycle that is more inclusive of country participation could foster better alignment of needs and allocation.

We also observe that allocation processes embody ethical principles, which we believe should be made more explicit. For instance, in its allocation process, Gavi prioritizes impact; in contrast GFATM prioritizes equity and stability, and the UN system institutions emphasize health need. Such discussion is important to promote fairness, and could be conducted on a participatory basis. At present, the types of support are constrained by politics, and none of the institutions are fully accountable to the countries or citizens they intend to benefit. As a personal conviction, we believe that actual and potential recipients have a right to know how allocation decisions are made and a right to representation in the process. We contribute this article as a step towards realizing these rights.

## Supplementary Data

Supplementary data are available at *HEAPOL* online.

## Supplementary Material

Supplementary Online Annex
