# Supplementary material for: Resource allocation processes at multilateral organizations working in global health
Source: Health Policy Plan. 2018 Feb 5;33(Suppl 1):i4–i13. doi: 10.1093/heapol/czx140 (PMC5886160; doi:10.1093/heapol/czx140)
Supplement: Supplementary Online Annex [file czx140_online_annex_nc.docx]

**Online Annex: Description of the resource allocation cycle in nine multilateral organizations working on global health**

**GFATM**

The GFATM supports countries by providing financing for programs addressing its three target diseases, or for developing health system capacities. Every four years, a new GFATM strategy is prepared by a working group created by the Board, the Strategic Investment and Impact Committee, led by the Executive Director and the strategy leads of the GFATM, as well as a team of independent consultants. Inputs from the Secretariat and working committees within the institution are considered. The process produces a document approved by the Executive Board of the GFATM, the highest governing body of the GFATM, which is composed of equal representation by implementers and donors. Subsequently, a conference is organized to mobilize resources to fulfil the objectives stated in the strategy. All donor pledges are collected and pooled together. Ninety-five percent of GFATM financing comes from national governments and the European Union. Contributions from private foundations, corporations and faith-based organizations contribute the remaining 5%. From the total pledges, an envelope is estimated for supporting country programmes for the three diseases and forwarded to the Board. The approved envelope is then split between the three diseases as follow: 50% for HIV/AIDS, 32% for Malaria and 18% for TB. In the latest replenishment, it was also agreed that $950 million will be set aside for countries to compete for additional funding for innovative projects (referred to as ‘incentive funding’), and $300 million for regional projects and special initiatives (GFATM, 2014).

The GFATM applies income-based and disease-burden based eligibility criteria. High-income countries are not eligible for GFATM financing, regardless of disease burden. Depending on income, further eligibility criteria are applied (e.g. focus of application, counterpart financing, G-20 membership). Subsequently, all eligible countries are assigned to a country band based on their disease burden and income level (Figure 3).

**Figure 3. Definition of country bands**


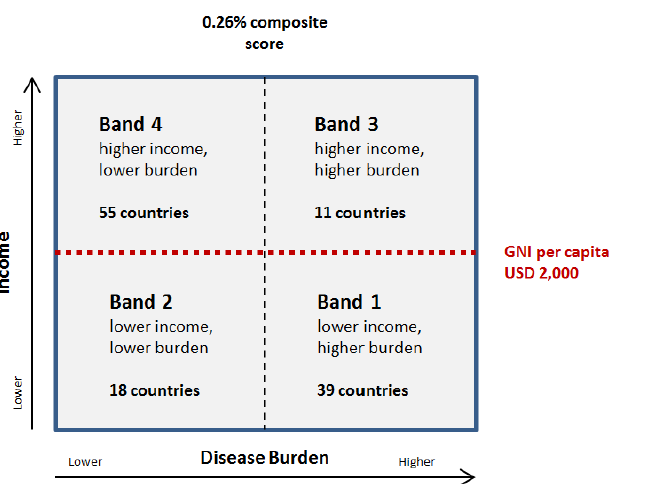


Source: (GFATM, 2014)

The Secretariat then runs an allocation formula for countries in Band 1, 2 and 3 (i.e., all countries except Band 4—higher income and low disease burden).^[[1]](#footnote-1)^ Each country is assigned a score calculated from ability to pay, which is derived from GNI per capita, and disease burden, which uses indicators developed by partner organizations such as the Stop TB Partnership and WHO. This score is used to determine relative shares for each disease for each eligible country. Each country receives one allocation figure that is the sum of the three disease allocations, and referred to as the indicative allocation.

Once this indicative allocation for all countries is determined, the Executive Board approves allocations to country bands, rather than individual country allocations. Following this, the Secretariat applies qualitative adjustments to countries to account for (i) past program performance; (ii) impact; (iii), increasing rates of infection; (iv) risk; (v) absorptive capacity; and (vi) other considerations (GFATM, 2014). Adjustments based on qualitative factors can change the allocations within a band, but they do not do so between bands. Additionally, allocations are bounded within two limits. The maximum per country allocation is capped at 7.5% of the total for the band. Also, for each country the variance from one allocation to the next is capped at 25%.

Once country allocations have been determined, decisions are communicated individually to countries. Countries then formulate a concept note detailing the proposed use of funds with the assistance of the Secretariat and external stakeholders. The Technical Review Panel, a group of experts, are then in charge of reviewing the proposals and help countries in submitting a quality proposal. Additional funding can also be obtained from the reserved incentive funding. In addition, countries can register an ‘Unfunded Quality Demand’, which is financed on a case-by-case basis by additional external contributions (requests are published online quarterly).

**Gavi, the Vaccine Alliance**

The GAVI strategy is set every four years by the Executive Board (assisted by Board Committees, composed of subject experts from Gavi and other organizations). The Board is composed of 28 members from international organisations. Permanent seats are held by UNICEF, WHO, the World Bank, and the Bill & Melinda Gates Foundation; others seats are occupied by independent experts, the vaccine industry representatives, governments and donor countries, and the CEO of Gavi’s Secretariat.

Gavi’s funding is collected through a replenishment process that begins after each new strategy is adopted. Gavi receives multi-year commitments from governments, foundations and private entities. Between 2000-2014, 69% of Gavi’s funding came from direct contributions from donor governments, the Bill and Melinda Gates Foundation and private donors. The remaining 31% was generated through innovative financing mechanisms (Advance Market Commitment and the International Finance Facility for Immunisation).

Only countries with a GNI per capita below US $1580 are eligible for Gavi support. In addition, countries should have an immunization rate for Diphtheria, Pertussis and Tetanus (DPT3) superior to 70% to be eligible for most vaccine support. This additional criterion is intended to ensure that basic routine vaccination infrastructure is in place. In 2014, Gavi supported 73 countries.

Gavi’s activities include vaccine programmes and technical support for health system strengthening. There are 11 vaccines in the current portfolio (2014–2018), which was defined in 2013. Gavi decides which vaccines will enter the vaccine investment strategy every four years. The vaccine investment strategy starts with a landscape analysis conducted by the World Health Organization, which identifies suitable vaccines with health benefits, high value for money, and fitting the strategic and programmatic specificities of Gavi’s work. Multiple consultations with Vaccine Alliance partners, in-country stakeholders and an independent expert committee are then organized to define the new vaccine portfolio. Gavi also provides support to health systems strengthening interventions (12% of Gavi disbursement to countries in 2014) (Gavi, 2015b).

Countries can apply for Gavi support in three application windows per year by sending an *Expression of Interest*, which is reviewed by the Independent Review Committee (IRC). Following approval of the expression of interest, countries develop an application, in line with the country’s strategy. These are reviewed by the Gavi Secretariat to ensure that the country has sufficient implementation capacity and adequate plans.^[[2]](#footnote-2)^ The Secretariat works closely with countries to strengthen the application. When ready, applications are presented at one of the several yearly meetings of the IRC and are either approved or returned with suggestions. For health systems strengthening applications, the support is calculated by multiplying the annual birth cohort by $5 for countries with a GNI per capita lower than $365 and by $2.5 for countries with a GNI per capita above $365. The minimum allocation is $3 million (Gavi, 2010).

If approved by the IRC, the Board allocates funding, provided there are sufficient resources to support all approved proposals. If there is not enough money, the Board decides which ones to fund immediately and which ones to fund in the next window. (We did not learn the criteria that are applied to adjudicate this issue). Funding allocation decisions are made by multiplying the size of the birth cohort by the price of the vaccine minus the level of co-financing. For newly approved vaccine programmes, a vaccine introduction grant is also included ($0.80 per child in the birth cohort for all vaccines except Human papillomavirus vaccine, for which countries receive $2.40 per girl in the birth cohort). For campaigns targeted to specific populations, a $0.65 per targeted person supplement can be disbursed to cover additional operational costs (Gavi, 2012b). Gavi support is also subject to co-financing that is linked to the country’s income. For instance, countries with an income below $1,045 in 2015 contribute $0.20 per vaccine dose (Gavi, 2015a). Once a program is started, support to countries continues until the country reaches an income threshold (when it ‘graduates’ from the pool of support).

**UNAIDS**

UNAIDS works to coordinate the HIV/AIDS-related work of 10 United Nations organisations and the World Bank (referred to as the Cosponsors). UNAIDS works on a 6-year cycle, which starts with the formulation of its global-level strategy, which defines a set of coherent activities and ‘results areas’ (e.g. access to prevention services for young people). These are produced to guide cosponsors’ resource allocation decisions and in-country work. The strategy is approved by UNAIDS’ governing body, the Programme Coordinating Board (PCB), which includes representatives of 22 countries, the 11 cosponsors, and 5 NGOs.. Representatives of cosponsors and NGOs do not have the right to vote. The PCB also works closely with the Executive Director, who is appointed by the UN Secretary General.

To support itself and HIV/AIDS work by the cosponsors, UNAIDS raises contributions from governments (3%), cosponsors (3%) and other partners (including private sector partners and foundations) (3%) (UNAIDS, 2016). In 2014, the United States, Sweden, Norway, the Netherlands and the United Kingdom were the main contributors of UNAIDS budget, together financing 66% of the total budget of the institution (UNAIDS, 2015).

About one-third of UNAIDS funds are allocated to cosponsors to strengthen their resource mobilization and programmes dedicated to the fight against HIV. The funding allocated to each cosponsor is determined based on “epidemic priorities, performance of the Cosponsors and the funds that each Cosponsor raises” (UNAIDS, 2011). The remaining two-thirds of resources are used for ‘development activities’ conducted by the UNAIDS Secretariat. This includes the work on advocacy, technical assistance, communication, partnerships, policy dialogue, accountability, and research through 85 country offices and six regional support teams.

UNAIDS plans its own work and allocates resources to partners using the Unified Budget, Results and Accountability Framework (UBRAF) (UNAIDS, 2011). UBRAF is defined through a consultative process involving the Cosponsors, UNAIDS Secretariat and other partners. UBRAF promotes coherence and coordination by specifying a division of labour among the cosponsors based on their expertise, existing work, and mandate. Because it includes activities by all of the cosponsors, it is also a mechanism for ensuring that all stated priorities are accounted for in the collective plan. Budget planning under UBRAF includes two categories of funding: UNAIDS core resources raised by the UNAIDS Secretariat, and non-core resources raised by cosponsors specifically for HIV/AIDS activities. These non-core resources are budgeted and accounted for on a biennial basis. UNAIDS core resources represent on average about 12% of the total funds spent by all Cosponsors on HIV/AIDS-related activities (UNAIDS, 2015).

**UNDP**

UNDP’s Administrator is appointed by the Secretary General of the UN. UNDP is overseen by an Executive Board that also has authority over UNFPA and UNOPS. This Board is composed of members representing 36 countries (serving on a rotating basis) and its bureau of 5 members representing each of the five UN regions, Every four years the Administrator of UNDP and the Executive Board supervise the development of a new strategy. From this strategy, a budget is developed and used to raise contributions from governments, and other multilateral organizations or private foundations. 74% of contributions to UNDP are earmarked, including the cost-shares provided by national governments for country office staff and operations. The remaining 26% of resources is provided as core resources (UNDP, 2013c). The United-States, GFATM, the European Union, Japan and the Global Environmental Facility were the top donors in 2014 (UNDP, 2015).

All countries are eligible for UNDP support, except those with high income, currently defined as GNI/capita of $12,475 or more (UNDP, 2013c). Support comes in the form of country policy support and service delivery, coordinated by its 170 country offices. UNDP also supports countries through technical cooperation and research.

Core resources are divided into three tiers under Targets for Resource Assignment from the Core (TRAC) (UNDP, 2013a):

- TRAC-1 (60% of regular funds) for programs
- TRAC-2 (31% of regular funds) a flexible fund to reward projects that are well performing, high impact, or innovative
- TRAC-3 (8% of regular funds) for conflicts or emergencies, such as natural disasters

TRAC-1 resources are allocated following a formula; TRAC-2 is allocated based on decisions made by the UNDP Administrator using qualitative factors on country program quality and impact, and TRAC 3 is demand driven as dictated by qualifying circumstances (UNDP, 2013c). Countries eligible for UNDP support are grouped based on GNI per capita^[[3]](#footnote-3)^:

- Low-income countries (average GNI per capita between 2008 and 2011 lower than $1280)
- Middle-income countries (average GNI per capita between 2008 and 2011 between $1280 and $12475)
- Net Contributing Countries (NCCs) with an average GNI per capita between 2008 and 2011 exceeding $12475 for at most two consecutive budget periods.

Only low-income countries and middle-income countries qualify for TRAC-1 funds, (NCCs are considered in a separate budget). Country shares are calculated using a formula with two variables: average 4-year GNI per capita and total population (both from World Bank data). Country allocations can be increased if the country is granted the status of Least Developed Countries (LDC), following the definition of the United-Nations. In addition, for countries transitioning from low-income to middle-income status, the difference between allocations made in two consecutive budget periods cannot exceed 45%.

The minimum allocation is set at $450,000 for low-income countries, LDC, and new middle-income countries with a UNDP country office. For lower middle-income countries (excluding those designated LDCs ), the minimum allocation for countries with an office is $350,000, and $50,000 for countries without a country office. Minimum allocation for upper-middle income countries (GNI per capita above $6,660 for the latest budgeting period) is $150,000 (UNDP, 2013b). No further criteria are taken into account for the calculation of the allocation.

UNDP’s work in countries is mainly defined through the UN Development Assistance Framework (UNDAF). The UNDAF includes a needs assessment used by all agencies and prepared jointly by the UN and the government. The UNDAF assessment is aligned with the national policy cycle, which facilitates coherence with national programmes. UNDP, based on the UNDAF assessment, formulates its own plans and budget for the country. Core resources are used to implement the plan, with additional resources sometimes available from the non-core, earmarked funds.

**UNFPA**

Every four years, UNFPA prepares a strategy and estimates the resources it needs for it. These are used as the basis for resource mobilization efforts. UNFPA draws its funding from voluntary contributions from governments, non-governmental organizations, foundations, and private institutions. 53% of UNFPA’s resources are earmarked to a specific activity or a thematic fund. The non-earmarked funds, 47% of total contributions, are pooled as core resources (often referred to as ‘regular resources’) (UNFPA, 2014).

UNFPA works with all countries, but its work in countries is based on an engagement framework that reflects country needs and domestic financing abilities. Accordingly, UNFPA conducts policy dialogue and advocacy in all countries, but only delivers services and interventions in low-income countries with needs judged high or very high, or lower-middle income countries with needs judged as very high. UNFPA’s country programs are based on the UNDAF.

Similar to other UN agencies, regular resources are allocated to country offices and are complemented by non-regular resources, including those earmarked for the country or earmarked for programs through thematic funds. Regular resources are allocated through three systems: (i) the Resource Allocation System (RAS), (ii) the global and regional programme, and (iii) institutional budget for management and development of UNFPA’s activities.

Most core resources are allocated to countries through the RAS, a point based system. The RAS uses six indicators to assess country needs: (i) skilled birth attendance for the poorest quintile of the population, (ii) proportion of met demand for modern contraception, (iii) adolescent fertility rate, (iv) maternal mortality ratio, (v) Gender Inequality Index, (vi) HIV prevalence among 15-24 year olds. In addition, countries with high risk for humanitarian crises and countries with high inequality rates might be weighted to receive more. Based on these indicators, countries are grouped in four categories according to need (highest, high, medium, low) and then further split according to four income categories, as shown in Table 2.

**Table 2. UNFPA’s matrix of country need and ability to finance, 2014–17 budgeting period**


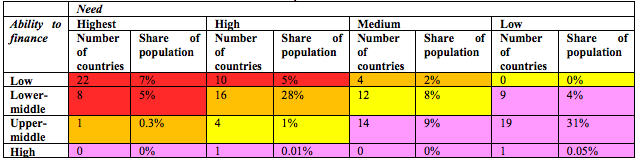


Source: UNFPA, 2014

The color coding in the table shows how UNFPA orders priority, from red, which designates countries with the highest need and lowest ability to finance, through pink, which indicates low need and substantial ability to finance. In the two intermediate levels, orange is higher than yellow.

All low and lower-middle income countries receive a minimum allocation of $500,000, and upper-middle and high-income countries receive a minimum of $300,000 per year (figures from the last budgetary period). The Executive Board decides on the level of resources that are allocated to each group. For 2016–17, it was decided that the red group (lower income, highest needs) would receive 59–63% of the envelope, the orange 20-22%, yellow 6-8% and the. pink group 9–13%..

**UNICEF**

UNICEF works on a four-year policy cycle, starting with the definition of a strategic plan and an integrated budget. Both documents are prepared and approved by the Executive Board. The Executive Board is composed of 36 members, which represent the member states of all five UN regions, and are elected to three year terms by the UN Social and Economic Council. The Bureau is composed by the President and four vice-presidents representing each region and oversees the work of the Executive Board.

UNICEF is funded with regular resources, also called core resources, which are not earmarked (26% of the total); and other resources**,** which are earmarked (74%). Primarily, these resources come from voluntary contributions and from fundraising by national committees. There are national committees in 36 developed countries. Formally, they are local NGOs; they raise funds by selling UNICEF products, and soliciting contributions from the private sector, civil society organizations, and individuals. In 2014, 43% of the UNICEF’s regular resources came from private sources (UNICEF, 2015b). Most of the balance came from donor countries and organizations. In 2014, the largest contributions were received from the United States, the United Kingdom, the European Commission, Norway, and Germany (UNICEF, 2015a).

UNICEF works with all countries where a need for UNICEF interventions is identified by the UN Development Assistance Framework (UNDAF). UNICEF does not apply income-related eligibility criteria, and envisions its work as ‘rights-based’, as stated in its latest strategy (UNICEF, 2016).

Based on the UNDAF, UNICEF develops a programme for each country and the Executive Board approves its corresponding budget. This agreed budget actually an indicative total for fundraising, and represents the maximum UNICEF expects to spend if it can raise enough. Core resources from are used to fund a portion of these budgets immediately; country offices raise additional resources that are earmarked to fund all or part of the balance. In general, the core resources are intended to support country office operations and resource mobilization efforts. However, core resources can be used to fund programme implementation.

The allocation of core resources to countries is calculated annually using a formula that dates to 1997 and was amended in 2008 (UNICEF, 2008). The allocation formula includes three criteria: under five mortality rates, GNI per capita, and the population of children aged 5 or less. Countries are classified in three groups according to the size of their child population. Under five child mortality rates are then used to calculate a weight ranging from 0 to 1 (0 corresponding to the country with the lowest child mortality rate and 1 to the country with the largest mortality rate). In the same manner, GNI per capita is used to calculate a weight ranging from 0 to 1, using a sliding scale, with the weight of 1 attributed to all low-income countries. Country scores are produced by multiplying these three weights, and the scores are used to calculate individual country allocation. The resulting allocation figures are then adjusted to fit within four parameters. First, the maximum difference in consecutive allocation periods is capped at 10%. Second, all countries with a country office receive a minimum allocation of $750,000, except for high-income countries. Third, UNICEF has a spending target of 60% of the total core resources on the Least Developed Countries (LDC), as defined by the United-Nations; the spending target for sub-Saharan Africa is 50% (UNICEF, 2012b).

Every year, a proportion of regular resources is also set aside for unplanned activities, such as funding programmes to address emerging opportunities, reward performance or compensate for important shortfalls in allocation. The Executive Director will make decisions over the use of these funds, and will be reported to the Executive Board.

**UNITAID**

Every four years, the Executive Board of UNITAID defines strategic objectives to fulfil its global mandate of improving access to prevention, detection and treatment tools for HIV/AIDS, malaria, and TB. These strategic objectives reflect emerging investment opportunities and the results of previous investments. The Executive Board is composed of 12 members, including one member appointed jointly by the five founding governments (Brazil, Chile, France, Norway and the United-Kingdom) and Spain, representatives of the African and Asia region, of civil society, of the WHO and of the constituency of foundations.

UNITAID was initially funded entirely by a solidarity levy on airline tickets, but by 2014 that source accounted for only 50% of the budget; multiyear commitments from nine national governments and two foundations comprised the balance. For instance, Norway allocates part of its carbon dioxide tax to UNITAID. In 2014, the main contributors were France, the United Kingdom, Norway, Brazil, the BMGF, and South Korea (UNTAID, 2015).

UNITAID provides grants to improve the access of drugs and supplies for HIV/AIDS, Malaria and TB, through the identification of a market gap and its corresponding solutions (in terms of drugs and supplies), and provide long-term grants to finance the development and provision of these solutions.. UNITAID only works through implementing partners, and does not work with governments or NGOs. Organisations are eligible for UNITAID support if they follow WHO’s operational principles for good pharmaceutical procurement and interagency guidelines, and if they have a permanent office or work relation with the country. Funding approved by UNITAID typically lasts between 3 and 5 years, but can be extended, subject to Board approval. UNITAID’s three largest partners are the Clinton Health Access Initiative, the GFATM, and UNICEF (accounting for about 70% of total funds committed between 2007–2012) (UNITAID, 2013).

UNITAID works on a rounds-based system, which starts with a call for proposals in a specific area, as in July of 2015: “to improve adult antiretroviral therapy in low and middle-income countries” (UNITAID, 2015). Organizations submit a letter of intent and, if approved, a full proposal. The main components of proposals are a description of equipment, supplies or drugs to be delivered, timeline, budget, organisational details, support letters, and policies on ethics, anti-discrimination, and the environment. The Proposal Review Committee and the Secretariat are responsible for reviewing and selecting proposals, and making recommendations to the board. Ultimately, the Executive Board decides funding agreements.

Implementing partners work with the Secretariat to decide how procured supplies and drugs will be allocated to countries. UNITAID doesn’t provide detailed guidelines for country allocations, but its constitution states that funds used to purchase health products should be spent as follows (UNITAID, 2011):

- at least 85% in low income countries
- no more than 10% in lower middle income countries
- no more than 5% in upper middle income countries

**World Health Organization**

The WHO is the intergovernmental specialised agency with the primary role to direct and coordinate international health within the United Nations’ system. The WHO’s main goal is to ‘support the attainment by all peoples of the highest possible level of health’ (constitution ref). The WHO is unique in its role in public health, as it performs important functions of disease surveillance, data collection, research, health promotion, and clinical advice to national public health institutions. The WHO also provides technical and operational support country in program implementation, provides grants to finance specific activities and technical assistance to countries. In 2015, the WHO had 194 member countries. The supreme decision-making governing body of the WHO is the World Health Assembly, which is composed of ministers from the member states. The World Health Assembly meets once a year to define and approve of the program of work, set major policy directions of the institution, and approve budget. The World Health Assembly also appoints the Director General of the WHO and the 34 members of the Executive Board. The main functions of the Executive Board are to give effect to the decisions and policies of the Health Assembly, to advise it and generally to facilitate its work.

The WHO is financed through assessed contributions (25%) paid by all member states, and voluntary contributions from countries, foundations, other UN and intergovernmental organizations, the private sector and NGOs (75%). All member countries contribute a fee (referred to as assessed contributions) the WHO budget depending on their level of income. Out of US$2.629 billion in 2014, US$929 million came from assessed contributions (World Health Organization, 2014). A vast majority of voluntary contributions (93%) comes in the form of earmarked contributions (World Health Organization). Negotiations between the WHO and its donors determine the use of these tied resources. The United States, Japan, Germany, United Kingdom and France are the largest contributors to the budget.

The WHO works with all member countries, although its level of engagement differs depending on the need and income of the country. WHO also works with partners ranging from governmental bodies to NGOs and civil society organizations. The headquarters, six regional offices as well as 147 country offices are responsible for implementing the programme of work voted by the World Health Assembly. Resources are allocated from the central budget to the regions based on a resource allocation model.

The WHO has recently gone through a reform of its resource allocation model of core resources. In 2014, a working group was formed with two member state representatives of each region to review the resource allocation process to the regions (World Health Organisation, 2015a). The following description applies to the current resource allocation framework, which was initiated in 2006 and will be used for the 2016-17 allocations.

Core resources are allocated to all Major Offices (regional offices and headquarters) every two years. Regional offices are primary point of technical advice for programmes and operations in individual countries, and have the capacity to take decisions on the use of the funds and disbursement to individual countries in line with the approved Programme Budget and based on agreed specificity identified by the donor. ). In this framework, allocations to the regions are made to cover technical cooperation costs for all countries in the region (referred to as ‘segment 1’ costs). Please refer to Financing of Programme Budget 2016-2017 at: <http://apps.who.int/gb/ebwha/pdf_files/WHA69/A69_46-en.pdf>

Under the current model, life expectancy and GDP per capita are the main inputs to the resource allocation calculation. Both indicators are scaled to have the same unit. The average of both scaled indicators is calculated per country (indicators are assigned equal weight). Based on this average, all countries are ranked and categorized in deciles. Countries in the top two deciles (with the lowest need) are not taken into account for the resource allocation to regions. Allocation to other deciles is made according to the table below (World Health Organisation, 2015b).

**Table X. Ranking per decile based on needs index and relative resource allocation**


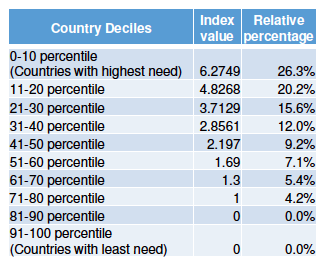


Source: (World Health Organisation, 2015b)

Country shares (or ‘weighting’ as referred to in WHO documents) are calculated by multiplying the needs index (based on the decile) to the country population. Currently, shares are adjusted for population using the adjusted log of population squared, which was chosen because the log of population reduces significantly the relative weighting of countries with very large populations.

A regional score is calculated by aggregating country weightings in a given region, and serves as a basis for regional allocations. Using this method, the allocation to the African region represents 43% of the total allocations in 2014-15, which is the largest and followed by the South East Asia Region (16%) (World Health Organisation, 2015a). In April 2016, the new funding model was adopted by a decision (reference: EB137(7)) prior to the 69^th^ World Health Assembly (World Health Organization, 2016). Under the new model, further indicators of needs (under 5 mortality and Non-Communicable Disease prevalence), poverty headcount, and indicators of access (Health workforce density, Political instability, and DTP3 coverage) will be included to calculate allocations for each country. Moreover, instead of ranking countries into deciles, the allocations will be calculated based on the composite score of the eight indicators of economic situation, needs and access (World Health Organization, 2016).

**World Bank/IDA**

Formally, IDA’s strategic cycle is distinct from those of most of the other reviewed institutions. At the beginning of each replenishment cycle, 52 IDA Deputies and 10 borrowing representatives discuss the experiences from the previous replenishment, and challenges, strategic directions, and special themes (climate change, gender issues, for instance) for the forthcoming period. IDA Deputies are appointed representatives of member states from both developed and developing countries. IDA’s replenishment is organized in three-year cycles. In the last replenishment^[[4]](#footnote-4)^, the largest contributions were made by the United States (2.7 million SDR), the United Kingdom (2.7 million SDR), Japan (2.4 million SDR), Germany (1.5 million SDR), and France (1.1 million SDR) (World Bank, 2013a). Total contributions amounted to 17.3 million SDR in the last replenishment. Loan repayment and income generated by other parts of the World Bank group—the International Bank for Reconstruction and Development (IBRD) and International Finance Corporation (IFC)—also provide resources for IDA.

IDA’s main activity is to offer concessional loans to low-income countries. Typically, IDA offers zero interest loans, but a grant component may be included depending on country’s risk of debt distress. County eligibility is defined by two elements: income—its threshold is adjusted yearly and in 2014 was set at US$1,205—and lack of access to capital markets. IDA countries must also be members of the International Monetary Fund (IMF). Exceptions are made in some cases for countries whose GNI per capita exceeds the income threshold but have poor credit ratings in the capital markets. For instance, Iraq is eligible for funding from IDA because it is considered a fragile state (World Bank, 2003). For similar reasons, Bosnia-Herzegovina retained IDA eligibility through 2015, even though it became a middle-income country many years earlier. Some transitioning countries also benefit from both the support of IDA and IBRD, and are referred to as blend countries. As of 2015, 77 countries are eligible for IDA support.

All IDA eligible countries are subject to a debt sustainability analysis and then classified as high risk, medium risk, or low risk of debt default. Since 2006, this rating has been the only consideration in calculating the grant element. Countries rated high risk receive their entire allocations as grants, while those at medium risk receive 50% of their allocations in grants. Countries with low risk and who do not borrow from IRBD receive their allocations as loans at zero interest for 25 or 40 years (World Bank, 2013b). IDA also provides technical support and does some program implementation.

IDA’s allocation decisions are made using the Performance Based Allocation (PBA), a formula that calculates ‘country shares’ based on indicators of quality of governance, population size, and GNI per capita. The PBA formula for calculating country shares can be written as follow (World Bank, 2014a):

**IDA country allocation** = f(Country Performance Rating^4^, Population, GNI per capita^-0.125^)


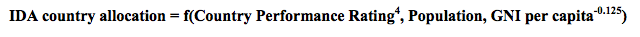


The Country Performance Rating is calculated based on the Country Policy and Institutional Assessment (CPIA) and the Country Portfolio Performance (PPR), which is the number of problems encountered by countries in previous IDA portfolio projects. The CPIA is a single indicator that assesses policies and institutional frameworks using indicators of (a) economic management; (b) structural policies; (c) policies for social inclusion and equity; and (d) public sector management and institution. The Country Performance Rating places a large emphasis on the CPIA.

A minimum base allocation for eligible countries is SDR 4 million a year; allocations are capped, as well, but there is not a single cap applied to all countries. Other adjustments are applied to define the final allocation figure for countries. For countries that receive the loan with a grant element, this grant element is discounted by 20%. These 20% are then reallocated to countries benefiting only from the support of IDA (non-blend countries) and blend countries with good credit ratings. Allocations for countries with an approved debt cancellation scheme will be reduced from the amount of the debt service in the following fiscal year (capped at 30% of the total PBA allocation for the period of consideration) (World Bank, 2010).

As of 2014, all countries seeking IDA funding have to undergo a systematic assessment to determine the main economic constraints and identify opportunities to promote growth for the poorest and abolish extreme poverty. This diagnosis is discussed between the Bank and the country in the development of the country partnership framework, which specifies how IDA fund wil be used. Countries facing exceptional circumstances or ‘turn-around countries’, for instance post conflict countries, can qualify for exceptional support, which negotiated on a case by case basis outside of the PBA system.

**Bibliography**

# Gavi. (2010). Health Systems Strengthening Resource Allocation. *GAVI Alliance Board Meeting (16-17 June).* Geneva: Gavi.

Gavi. (2012b). *GAVI Alliance Vaccine Introduction Grant and Operational Support for Campaigns Policy.* Geneva: GAVI.

Gavi. (2015a). *Review of GAVI's Co-financing Policy, Report to the Board 10-11 June 2015.* Geneva: GAVI.

Gavi. (2015b). *Gavi, The Vaccine Alliance 2014 Annual Financial Report.* Geneva: Gavi, The Vaccine Alliance.

GFATM. (2014). *Overview of the Allocation Methodology (2014-2017).* Geneva: GFATM.

UNAIDS. (2011). *UNAIDS 2012-2015 Unified Budget, Results and Accountability Framework.* Geneva: UNAIDS.

UNDP. (2013a). *Annex to DP/2013/41 ("UNDP integrated budget estimates for 2014-2017"): Methodology.* New York: UNDP.

UNDP. (2013b). *Funding of differentiated physical presence.* New York: UNDP.

UNDP. (2013c). *UNDP integrated budget estimates for 2014-2017, Report of the Administrator.* New York: United-Nations.

UNDP. (2015). *Top Contributors*. Retrieved 01 03, 2016, from undp.org: <http://www.undp.org/content/undp/en/home/ourwork/funding/top-contributors/>

UNAIDS. (2015). *UNAIDS Unified Budget, Results and Accountability Framework 2016–2021 (Agenda Item 4).* Geneva: UNAIDS.

UNAIDS. (2016). *Total contributions 2015 (in US dollars).* Geneva: UNAIDS.

UNFPA. (2014). *Funds and funding*. Retrieved 12 01, 2015, from UNFPA: <http://www.unfpa.org/funds-and-funding>

UNICEF. (2008). *UNICEF Executive Board Decision 2008/15, Report on implementation of the ‘modified system for allocation of regular resources for programmes’ approved by the Executive Board in 1997.* New York: United Nations.

UNICEF. (2012b, 08 12). *Report on Implementation of Modified System for Allocation of Regular Resources for Programmes.* Retrieved 07 01, 2015, from <http://www.unicef.org/about/execboard/files/RR_Allocation_Presentation_to_Informal_29Aug2012.pdf>

UNICEF. (2015a). *Annual Report: 2014.* New York: UNICEF.

UNICEF. (2015b, June 19). *Regular Resources*. Retrieved November 20, 2015, from UNICEF: <http://www.unicef.org/publicpartnerships/66662_66850.html>

UNICEF. (2016, 01 23). *Human Rights-based Approach to Programming*. Retrieved 02 26, 2016, from UNICEF: <http://www.unicef.org/policyanalysis/rights/>

UNITAID. (2011). UNITAID Constitution.

UNITAID. (2013). *Strategy 2013-2016.* UNITAID.

UNITAID. (2015, 06 3-4). *UNITAID.* Retrieved 02 26, 2016, from UNITAID Resolution 4: Area for Intervention: improvement of adult antiretroviral therapy in low and middle income countries: <http://www.unitaid.eu/images/Resolutions/eb22/R4_EB22_Areas_for_Intervention_ARV.pdf>

UNTAID. (2015). *Audited Financial Report. For the year that ended on the 31 December 2014.* Geneva: UNITAID

World Bank. (2003). *IDA’s Role in Potential World Bank Lending to Iraq.* Washington: World Bank.

World Bank. (2010). *A Mechanism for Mitigating the Allocation Impact of MDRI Netting Out.* Office of the President. Washington D.C.: World Bank.

World Bank. (2013a). *Contributions to the Sixteenth Replenishment.* Retrieved 01 02, 2016, from World Bank: <http://www.worldbank.org/ida/papers/IDA16_Donor_Contributions_Table_1.pdf>

World Bank. (2013b). *IDA’s Long Term Financial Capacity and Financial Instruments International Development.* IDA Resource Mobilization Department (CFPIR). Washington D.C.: World Bank.

World Bank. (2014a). *IDA’s Performance-Based Allocation System for IDA17 (Annex 2).* Washington D.C.: World Bank.

World Health Organization. (n.d.). *About WHO - Voluntary Contributions*. Retrieved 04 27, 2016, from The World Health Organization: <http://who.int/about/finances-accountability/funding/voluntary-contributions/en/>

World Health Organization. (2014). *Programme Budget 2014-15.* Geneva: World Health Organization.

World Health Organisation. (2015a). *Strategic budget space allocation.* Director-General. Geneva: United Nations.

World Health Organisation. (2015b, January 21). Information on Methodology for Segment 1.

World Health Organization. (2016). *Financing of Programme budget 2016 - 2017: Strategic budget space allocation.* Geneva: World Health Organization.

1. Countries in Band 4 receive collectively 7% of the total envelope and a separate process is applied based on population size. [↑](#footnote-ref-1)
2. Gavi assesses capacity and plans in many ways, including the history of DPT3 coverage rates, existence of a national technical advisory group with clearly defined roles, the alignment of the proposal with national health objectives, the national priority of the disease in question, analysis of barriers to access and utilization by women and by various socio-economic groups, detailed activity plan and budgets covering procurement mechanisms, staff remuneration, financial management and other factors, and reporting and data quality capacities (GAVI, 2014).” [↑](#footnote-ref-2)
3. Thresholds are for the period 2014–2017 [↑](#footnote-ref-3)
4. World Bank uses the Standard Drawing Rate (SDR) as a currency for its allocations. SDR is calculated by the IMF by summing the values in U.S. dollars, based on market exchange rates, of a basket of major currencies (the U.S. dollar, Euro, Japanese yen, and pound sterling). See <https://www.imf.org/external/np/fin/data/rms_sdrv.aspx> for conversion rates [↑](#footnote-ref-4)
